# PICU treatment of 3 cases of pediatric thyroid storm: Case series and literature review

**DOI:** 10.1097/MD.0000000000033447

**Published:** 2022-04-07

**Authors:** Xinyao Li, Jun Chen, Zhuo Li

**Affiliations:** a Department of Emergency/Critical Medicine, Children’s Hospital of Nanjing Medical University, Nanjing, Jiangsu, China.

**Keywords:** case reports, pediatrics, thyroid storm

## Abstract

Thyroid storm (TS), also known as thyroid crisis, is a life-threatening condition that involves multiple organ dysfunction and high mortality due to uncontrolled hyperthyroidism. TS in children is extremely rare, early diagnosis and treatment can significantly improve the prognosis of the children.

**Patient concerns:**

Three female children who diagnosed as “thyroid storm” were admitted to Pediatric Intensive Care Unit (PICU). One of them had a family history of hyperthyroidism and others had infection factors induced TS. They presented with characteristic manifestations of TS and were evaluated with Burch-Wartofsky Point Scale (BWPS) hyperthyroidism score.

**Diagnoses:**

Three cases showed that free triiodothyronine 3 (FT3) and free triiodothyronine 4 (FT4) were increased and Thyroid-Stimulating-Hormone was significantly decreased, which were characteristic in hyperthyroidism. They presented with characteristic manifestations of TS and were evaluated with BWPS hyperthyroidism score.

**Interventions:**

All the cases were given antithyroid drugs (ATDs) for treatment. In addition, 1 of them underwent therapeutic plasma exchange (TPE) after transferring to PICU.

**Outcomes:**

One of the cases was declared dead and others were survived.

**Lessons:**

TS should be identified timely and treated early. Further studies are needed to determine the diagnostic criteria and scoring system for TS in pediatric.

## 1. Introduction

Thyroid storm (TS) is a rare and life-threatening disease, first described as “crisis of exothalmic goiter” by Lahey FH et al in 1928 and suggested that it should be recognized and treated early; current studies in adults indicate that the incidence of TS is 0.2 per 100,000 people per year in Japan, with a prevalence of 0.22% of hyperthyroidism and an overall mortality rate of 10.7%. TS is more common in women, with a male-to-female ratio of 1:3.^[1,2]^ Sporadic cases have been reported in pediatrics.^[3,4]^ At present, most of the case reports of TS are in adults and rare in pediatrics. This article reports 3 cases of children, introduces the diagnosis and treatment methods, provides a reference treatment for children with TS.

## 2. Case presentation

Case 1, a female child aged 9 years and 2 months (137.5 cm/29.5 kg), was admitted to the hospital on February 14, 2021, with “thickened neck for more than 5 months,” and it was aggravated with palpitations and sweating for 1 more day. The child had recently been admitted to the local hospital with increased sweating, occasional palpitations, and no significant change in personality. However, the treatment was irregular, and the medication was intermittently stopped. Her mother had been diagnosed with Hashimoto thyroiditis for 4 to 5 years and was currently on oral treatment with Eugenol; her uncle had a history of hyperthyroidism and was off medication. On arrival, vital signs showed temperature of 36.3°C, blood pressure (BP) of 116/68 mm Hg, respiratory rate (RR) of 24 beats/min and heart rate (HR) of 102 beats/min. She was in normal state of consciousness, and had II degree enlargement of the thyroid gland, mild tremor of hands, but no pathological reflexes elicited. Two days after admission, the child developed high fever, increased HR and RR, confusion, pale face, blunted pupil light reflex, decreased BP, and a Burch-Wartofsky Point Scale (BWPS) hyperthyroidism score of 75^[5]^ (temperature ≥ 40 °C, HR ≥ 140 beats/min, moderate central nervous system symptoms), which was diagnosed as “thyroid storm.” She was then transferred to the Pediatric Intensive Care Unit (PICU) on the 16th (Table 1).

**Table 1:** Clinical characteristics of children with thyroid crisis after PICU.

Variable	Case 1	Case 2	Case 3
Age (mo)	110	43	102
Temperature (°C)	39.9	37. 1	37.4
Heart rate (bpm)	161	148	134
Respiratory rate (bpm)	43	39	31
MAP (mm Hg)	40	57	61
Coma (Y/N)	Y	N	N
Sepsis (Y/N)	N	N	Y
Vasoactive drugs (Y/N)	Y	N	N
Vasoactive drug score	25	-	-
Shock (Y/N)	Y	N	N
Organ dysfunction (Y/N)	Y	Y	Y
Specific organs	Respiratory; heart	Heart; liver	Heart; liver
Hyperthyroid crisis score	85	25	35
MV (Y/N)	Y	N	N
CRRT (Y/N)	N	Y	N
Antithyroid drug therapy	Y	Y	Y
Propylthiouracil	50 mg po q8 h×1 d	100 mg po q8 h ×3 d	150 mg po q8 h×3 d
Compound iodine solution	–	–	–
Propranolol	7.5 mg po q8 h ×1 d	5 mg po q8 h ×3 d	10 mg po q8 h × 1 d; 7.5 mg po q8 h × 3 d
Glucocorticoids	50 mg q8 h ×1 d	50 mg q6 h ×3 d	100 mg q8 h × 2 d; 50 mg q8 h × 1 d
Others	–	Methimazole 10 mg qd × 3 d	Methimazole 10 mg Bid × 3 d
Prognosis	Dead	Survived	Survived
Length of stay in PICU	<24 h	3 d	6 d

CRRT = continuous renal replacement therapy, MV = mechanical ventilation, PICU = pediatric intensive care unit, Y/N = Yes/No.

**Figure 1.**
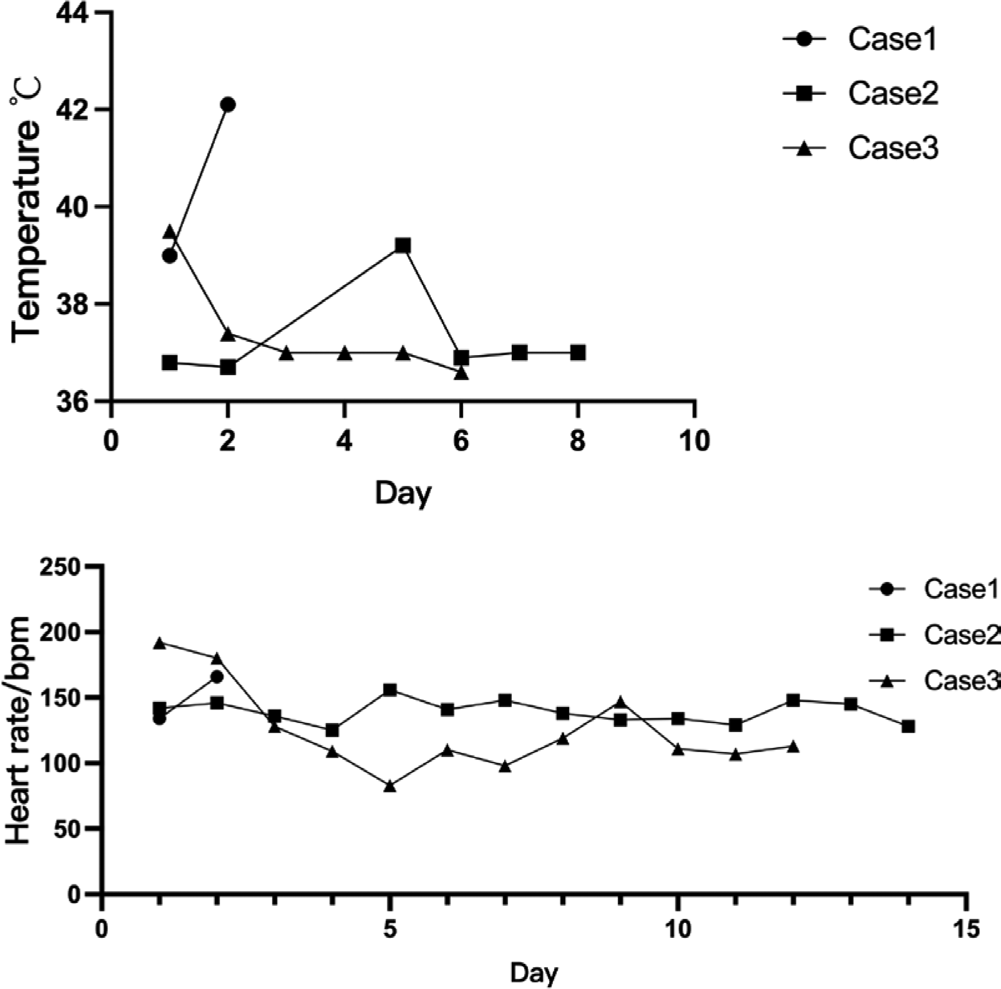
Temperature and HR of 3 cases after admission. HR = heart rate.

The changes in the temperature and HR after admission are shown in Figure 1, the results of multiple rechecking of thyroid function and anti-thyroid antibodies are shown in Table 2.

**Table 2:** Thyroid function and related antibody indexes of 3 patients.

Case	T3 ^(nmol/L)^	T4 ^(nmol/L)^	FT3 ^(pmol/L)^	FT4 ^(pmol/L)^	^TSH (μIU/mL)^	^TG-Ab (IU/mL)^
Case 1						
1	2.52	97.23	9. 11	22.32	^<0.005^	1680
2	2. 16	109.6	6.61	20.3	^<0.005^	2079
Case 2						
1	^>10.0^	289.4	48.87	^>100.0^	^<0.005^	440.2
2	4.89	216.4	24.37	63.24	^<0.005^	410.8
3	1.81	157.8	6. 11	37. 14	^<0.005^	407
4	3.64	115.3	12. 12	21.23	^<0.005^	384. 1
Case 3						
1	4.49	186.8	18. 18	91.94	^<0.005^	653.7
2	1.52	183.4	4.62	57.32	^<0.005^	591. 1
3	3.4	213. 1	13.24	76.36	^<0.005^	588.3
4	5.88	186. 1	24. 14	54.29	^<0.005^	503.6
Reference	1.43–3.55	77. 1–178	3.88–8.02	12.5–21.5	0.60–4.84	0–37

FT3 = free triiodothyronine 3, FT4 = free triiodothyronine 4, TSH = Thyroid-Stimulating-Hormone.

After case 1 was transferred to PICU, an emergency bedside electrocardiogram showed sinus tachycardia and cardiac ultrasound showed cardiac insufficiency. Propylthiouracil (PTU) 50 mg q8 h orally, propranolol 7.5 mg q8 h orally and hydrocortisone 50 mg q8 h by infusion were given to control TS, norepinephrine was started at 0.1 μg/kg/min titrated to maintain BP. After 4 hours, the pupils became dilated and the BP decreased, so the pump speed of norepinephrine was gradually increased. Simultaneously, dobutamine and dopamine were added at a titration of 5 μg/kg/min. The child BP remained unstable and the HR progressively decreased. During this period, cardiac pulmonary resuscitation, 1:10, 000 epinephrine 0.1 mL/kg by intravenous injection for 11 times, and cardiac electrical resuscitation (energy 30 J) twice are conducted to rescue, but the child still failed to maintain vital signs. After 3 hours of continuous resuscitation, the child was declared dead after adequate communication with the family.

Case 2, a female child aged 3 years and 7 months old (111.3 cm/19 kg), was admitted to the hospital on April 14, 2021, with “diarrhea for 4 days and fever for half one day.” The parents found that the child was unresponsive, with slight tremors of the extremities, and that the child had excessive sweating in the past, without personality changes. On arrival, vital signs showed temperature of 36.8°C, BP of 109/53 mm Hg, RR of 36 beats/min and HR of 136 beats/min. She was in normal state of consciousness, had slightly protruding eyes, II degree enlargement of the thyroid gland, and mild tremor of the hands. After admission, the patient was considered to be “hyperthyroidism” due to abnormal thyroid function and was transferred to the department of endocrinology for further treatment. Five days after admission, the child developed high fever, diarrhea, increased HR and RR, depression, flushing, and a BWPS hyperthyroidism score of 55 (temperature of 39.3°C, HR ≥ 140 beats/min, diarrhea), which was diagnosed as “thyroid storm.” She was transferred to PICU on the 19th (Table 1).

The changes in the temperature and HR after admission are shown in Figure 1, the results of multiple rechecking of thyroid function and anti-thyroid antibodies are shown in Table 2. The basal metabolic rate of case 2 in 3 days was 66%-54%-87%.

Case 2 underwent therapeutic plasma exchange (TPE) for 1 time after transferring to PICU. Besides TPE, she was given PTU 100 mg q8 h orally, methimazole (MMI) 10 mg qd orally, propranolol 5 mg q8 h orally and hydrocortisone 50 mg q6h by infusion for 3 days. After treatment, her vital signs can be maintained as normal with no limb tremors, and thyroid function is better on recheck than before. She was transferred to the general ward on the 22nd for continued treatment. PTU was discontinued 1 day later, hydrocortisone was discontinued 2 days later, and prednisone tablets 10 mg qd were added orally, which was reduced by half to 2.5 mg qd every other day and then discontinued, she was discharged on April 29, 2021.

Case 3, a female child aged 8 years and 6 months (130 cm/28.5 kg), was admitted to the hospital on April 22, 2022, with “abdominal pain, diarrhea with vomiting for more than 1 day and fever for half a day.” The child had an increased HR during the course of the disease. The child did not have excessive sweating or personality changes in the past. On arrival, vital signs showed temperature of 37.4°C, BP of 129/78 mm Hg, RR of 35 beats/min and HR of 142 beats/min. She had slightly shortness of breath, no obvious enlargement of the thyroid gland, and low heart sounds on auscultation. Two hours after admission, the child developed hyperthermia, increased HR and RR, confusion, delirium, and a large amount of dilute watery stool, with a BWPS hyperthyroidism score of 95 (temperature 39.5°C, HR ≥ 140 beats/min, diarrhea, delirium), which was diagnosed as “thyroid storm” (Table 1).

The changes in the temperature and HR after admission are shown in Figure 1, the results of multiple rechecking of thyroid function and anti-thyroid antibodies are shown in Table 2. The basal metabolic rate of case 2 in 3 days was 40%-39%-44%.

Case 3 was treated with PTU 150 mg q8 h orally for 2 days (5 mg/ kg) after 18 hours of admission, and was switched to MMI 10 mg qd orally for 3 days, propranolol 7.5 mg q8 h orally for 3 days/propranolol 10 mg q8 h orally for 1 day, and hydrocortisone 100 mg q8 h orally for 2 days after discontinuation of the drug, after treatment her vital signs could be maintained as normal. She was transferred to the general ward on the 27th for continued treatment. The child was given MMI 10 mg bid orally for 5 days and propranolol 7.5 mg q8 h orally for 3 days, and discharged on May 3, 2021.

## 3. Discussion

TS is a rare and life-threatening disease in pediatrics, which needs early diagnosis and timely treatment. This article reports 3 cases treated in PICU, all the cases were female girls and developed rapidly.

The etiology and pathogenesis of TS are not clear. The most common underlying cause is Graves’ disease and the most common causative agent seems to be various infections.^[6]^ In our report, case 1 had an insidious onset, combined with a family history of hyperthyroidism and history of noncompliance to medications. She was not regularly reviewed and followed up. Thyroid function was not effectively controlled, resulting in rapid progression to TS after admission. Case 2 showed an increase in white blood cells, mainly neutrophils, and case 3 showed a significant increase in C-reactive protein, therefore, 2 cases were considered early co-infection induced TS.

The common manifestations of TS are a sudden worsening of the preexisting hyperthyroid manifestations, and some cases may also present with gastrointestinal symptoms. In addition, TS may manifest with multiple organ dysfunction syndrome. Case 1 presented with characteristic manifestations of TS such as hyperthermia and increased HR/RR, later combined with multiple organ dysfunction syndrome, such as confusion, unstable BP requiring vasoactive drugs and respiratory failure, the electrocardiogram showed sinus tachycardia, which is the most common type of arrhythmia in TS.^[7]^ Cases 2 and 3 both started with diarrhea and showed characteristic manifestations, thyroid function showed that free triiodothyronine 3 (FT3) and free triiodothyronine 4 (FT4) were increased and Thyroid-Stimulating-Hormone was significantly decreased, which were characteristic in hyperthyroidism. However, there is no evidence that FT3/FT4 increases in varying degrees can be used to differentiate between hyperthyroidism and TS. Swee du, S et al^[8]^ showed that gastrointestinal and cardiovascular symptoms were most common in adult patients, pyrexia of ≥ 38.2°C was seen in 25% of adult patients. In this report, all patients presented with hyperpyrexia of ≥ 39.1°C and tachycardia of ≥ 140 beats/min. Otherwise, temperature of case 1 was 42.1°C, which was higher than other cases (39.2°C and 39.5°C). Thus, children are more likely to develop hyperpyrexia than adults, and higher temperature may predict worse prognosis.

TS lacks clear and uniform diagnostic criteria, and the diagnosis is mainly based on clinical manifestations, while early diagnosis is the key to treatment. In 1993, Burch et al proposed the BWPS,^[1]^ which is widely used for clinical diagnosis of TS. This criterion is highly sensitive and therefore has a high rate of false positives, and the mechanism of assigning scores to each clinical manifestation is complex and has not been validated. Based on this, the JTA proposed a new diagnostic criterion. They divide TS into confirmed TS (TS1) and suspected TS (TS2), and removed the scoring system from quantifying the diagnosis of TS.^[3]^ The combination of the 2 criterion is recommended clinically to improve the diagnostic accuracy. There is no uniform scoring criteria in pediatrics, but criteria in adult can provide reference and assessment value for the diagnosis and treatment of TS in pediatrics. The BWPS plays an important role in 3 cases, so the adult TS diagnostic criteria can be used by pediatric internal medicine and critical care physicians in conjunction with clinical practice.

**Figure 2.**
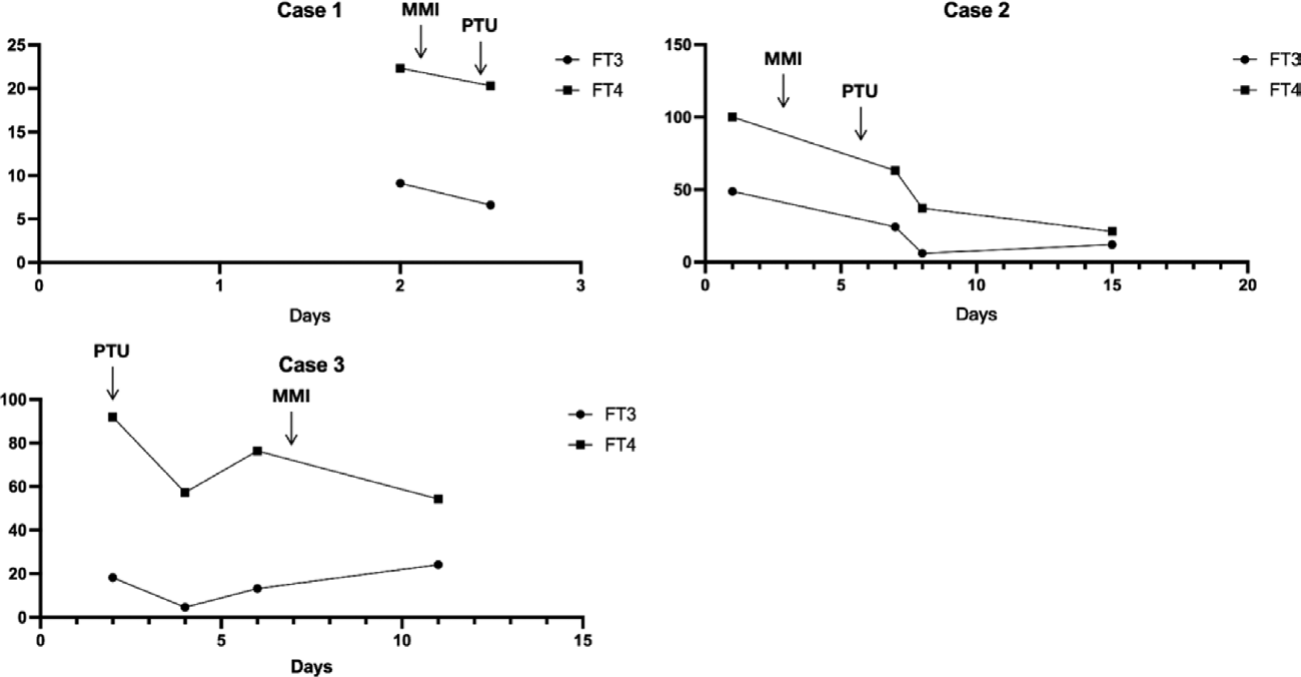
The thyroid hormone levels (FT3/FT4). FT3 = free triiodothyronine 3, FT4 = free triiodothyronine 4.

The clinical progression of TS is very rapid and requires timely and comprehensive treatment. A guideline was published in Japan in 2016 to guide the treatment of TS^[9]^: a combination of ATDs (MMI or PTU), inorganic iodides, glucocorticoids, β-AAs, and antipyretics should be used to ameliorate TS and its adverse effects on multiple organ systems; TPE; the Individualized treatment regimens are implemented for patients. All 3 cases used ATDs to inhibit thyroxine synthesis at an early stage and achieved good clinical results with a clear trend of visible decrease in FT3/FT4 (Fig. 2), in addition, case 2 underwent TPE once after transferring to PICU because of ineffective control of hyperthyroid symptoms with MMI. In 2019 the American Society for Apheresis has included TS as a Class II indication for TPE, which is applied to significantly reduce FT3\FT4\TT3\TT4 levels when first-line therapy is not effective.^[10]^

## 4. Conclusion

TS is a very rare disease in pediatrics, lacking accepted diagnostic criteria, and once present, it progresses rapidly with high mortality. There is a lack of uniform diagnostic criteria for children, and the combination of adult morbidity characteristics and means of treatment, combined with the currently available rescue cases reveals that early recognition of common symptoms, early diagnosis and timely treatment are the main measures to improve the prognosis of children. Therefore, when a child with hyperthyroidism clinically presents with high fever and sweating, mental irritability, increased HR/RR, with or without diarrhea and vomiting, and cardiac insufficiency, the diagnosis of TS should be considered, and the comprehensive rescue measures of applying ATDs, propranolol, and glucocorticoids can effectively alleviate and reduce mortality. Therefore, clinical pediatrician should identify TS timely and treat it accordingly. With the accumulation of clinical experience, it is expected that the diagnostic criteria and scoring system for TS in pediatrics will be available.

## Author contributions

**Conceptualization:** Zhuo Li.

Supervision: Zhuo Li.

Writing – original draft: Xinyao Li.

Writing – review & editing: Jun Chen.
